# Hybrid Effectiveness-Implementation Trial of a Pilot Rental Subsidy Program at an Area Agency on Aging.

**DOI:** 10.1093/geroni/igaf122.4363

**Published:** 2025-12-31

**Authors:** Anthony Traver, Katie Calhoun, Marisa Sheldon

**Affiliations:** University of Kentucky, Lexington, Kentucky, United States; The Ohio State University, Columbus, Ohio, United States; The Ohio State University, Columbus, Ohio, United States

## Abstract

The prevalence and severity of housing insecurity among older adults in the U.S. are rising and leading to unmet needs, premature nursing admissions, and homelessness. Across the country, area agencies of aging (AAA) are developing new programs and partnerships to address housing insecurity in later life. In 2024, our research team partnered with an AAA to develop a shallow rental subsidy program that provided $330 of rental assistance a month for 12 months to housing-insecure home and community-based (HCBS) clients and evaluated the program through a hybrid effectiveness-implementation trial. We assessed program effectiveness through a 12-month mixed-methods randomized controlled trial with 40 HCBS clients that focused on housing, health, and service utilization outcomes. Treatment effects were estimated using longitudinal analysis of covariance and mixed with qualitative data using the primary data analysis integration procedure. Implementation facilitators and barriers were assessed through surveys and focus groups with AAA staff and interpreted using the Consolidated Framework for Implementation Research (CFIR). Mixed-methods results demonstrated positive program impacts on well-being by alleviating financial distress associated with rising rental costs. Qualitative results that reflected positive impacts on housing security diverged from quantitative results of a null effect. Implementation results revealed a strong tension for change within the AAA that facilitated implementation, but a lack of program compatibility and limited information dissemination were barriers. Our results demonstrate that shallow rental subsidies can be a meaningful complement to HCBS for housing-insecure AAA clients and identify key agency-level features that can promote implementation within the AAA context.

